# An emotionally loaded subject – parents experiences when CHC-nurses introduce a conversation related to their child being overweight

**DOI:** 10.1186/s12889-024-20696-2

**Published:** 2024-11-20

**Authors:** Marie Fridolfsson, Anna Olsson, Jenny Ericson, Catrin Borneskog, Catrin Borneskog Sinclair

**Affiliations:** School of Health and Welfare, Dalarna University, Falun, 791 88 Sweden

**Keywords:** Children, Child health care nurse, Experience, Overweight, Parents, Qualitative

## Abstract

**Background:**

Children with a raised BMI are a major public health challenge in the world that can cause serious consequences for children’s physical and mental health. Child health care nurses’ responsibility is to promote health and prevent ill-health in children, but because being overweight is stigmatized CHC-nurses find it difficult to talk to the parents about concerns over the children’s weight, in fear of losing their trust.

**Aim:**

The purpose of this study was to describe parents’ experiences of when CHC-nurses introduce a conversation related to their child being overweight.

**Method:**

This study was conducted via semi-structured interviews with nine parents and the data was analyzed using qualitative content analysis.

**Results:**

The result showed that being overweight in childhood is a sensitive topic, and this is presented as four categories *A hard time coping*,* A desire to protect the Children*,* Changes of Parental Perspective* and *Trust - a mediating factor.* This is a sensitive topic for the parents, who have a hard time accepting the situation and place the blame on themselves, and the nurse’s approach and relationship with the parents is of great importance for the outcome of the conversation.

**Conclusions:**

Parents seem to have another perspective on health and weight than the healthcare providers, and both parents and CHC-nurses might benefit from an increased understanding of one another’s view. Parents need to gain knowledge about how healthcare providers assess children’s growth, and the CHC-nurses need to be aware about how to be responsive to the parents and allow them time to process the information. The focus should be on healthy lifestyle choices regardless of weight.

## Introduction

Overweight and obesity in children have increased worldwide during the last 40 years. This increase can be seen as a result of socio-cultural and societal changes associated with urban development, such as a sedentary lifestyle and easy access to energy-dense nutrient-poor food [1, 2]. Being overweight increases the risk for cardiovascular diseases, cancer, and type 2 diabetes. Being overweight in childhood is associated with an increased risk of fractures, hypertension, insulin resistance and obesity in the future, as well as disability and premature death [2]. Childhood overweight can also lead to psychosocial consequences such as depression, loneliness, exclusion, discrimination, and low self-confidence, as well as getting bullied [3]. Being overweight is stigmatized and those affected are often assigned negative attributes such as being lazy, clumsy, unintelligent, undisciplined, and having poor hygiene [4], even among preschool children [5].

In Sweden all children up to six years old are offered health check-ups at publicly financed Child Health Care (CHC) centers where CHC-nurses work to promote children’s health and support their families. Nearly 100% of the families attend, and during routine check-ups CHC-nurses assess children’s development and track their growth and give customized advice to the families when needed [6]. CHC-nurses find it hard to identify and assess overweight in children [7] and they find it difficult to give support and advice to these families [8, 9] and are afraid to hurt the parent’s feelings [10]. At the same time parents tend to feel offended when informed about their child’s overweight, and doubt whether it’s true [11, 12]. The conversation can be perceived as either empowering or provoke resistance depending on how the subject is approached [11]. Parents seem to underestimate their children’s weight status [13, 14] as well as the risks associated with being overweight in childhood [15, 16] and many think that it will resolve by itself [13, 17]. Studies investigating the weight stigma indicate that parents often feel judged and blamed for their children being overweight [18, 19] and some fear that taking actions to lose weight might affect the children negatively [11].

Being overweight in childhood is a growing issue with major health consequences. As a CHC-nurse, the tasks include working to promote health and prevent ill-health in children, but it is perceived as difficult to talk about a child being overweight with the parents. There is a fear that the conversation will damage the caring relationship and there is a concern regarding how the information will be received. Thus, an increased understanding of the parents’ perspective on the conversation concerning the child’s weight is needed so that these conversations can be carried out as well as possible.

### Aim

The aim of this study was to describe parents’ experiences of when CHC-nurses introduce a conversation related to their child being overweight.

## Method

A qualitative design with an inductive approach was used since it was a suitable method to answer the aim and create a new understanding for a phenomenon [20]. Data were collected through semi-structured interviews which made it possible for the parents to speak freely about their experiences within the context of the subject [20]. This study took place in Sweden.

### Participants

Participants were recruited through advertising in social media (Facebook) during September and October 2022. The advertisement sought parents who had experienced a conversation about their child being overweight with their CHC-nurse within the past two years and while their child was between 2 and 5 years old. Those wanting to participate were asked to announce their interest via email, and a total of 15 parents responded. An information letter about the study as well as an informed consent form was emailed, and parents were asked to send the signed consent back to the research team if they still wanted to participate. Five stopped responding or dropped out and one was excluded due to not meeting the inclusion criteria, resulting in a total of nine participants in this study.

All participants were Caucasian females between the ages 23 and 45, and six of them had higher education.

### Data collection

A semi-structured interview guide was created and tested in a pilot interview [20], that led to some adjustments where one main question was changed into a follow up-question, ending in a total of five main questions and five follow-up questions. The interviews were carried out continuously during the recruitment period and the first question asked to all participants was “Tell me what the CHC-nurse did say about your child’s weight?” followed by the follow-up question “How did it feel?“. All the interviews were conducted in Swedish via Zoom and lasted between 14 and 29 min. The two first authors were both present in all the interviews where one asked the questions and the other one listened and then took turns leading the interviews. Audio recordings were made of the interviews while Microsoft Word’s dictation function dictated in real time. Directly after each interview the two first authors listened to the audio-files while reading and correcting the texts the program made. When all interviews were transcribed, they were individually listened to and read through again by the first authors to make sure everything was transcribed correctly. One participant was then contacted again to follow up some questions. The pilot interview was not included in the data.

### Analysis

The material was analysed using qualitative content analysis with an inductive approach, by Elo and Kyngäs [21]. After reading the transcriptions multiple times the two first authors individually coded the transcriptions. Although, there was some pre-understanding regarding the subject since the first authors had read discussions on social media where parents expressed negative opinions about the healthcare system regarding children and weight. The first authors were aware of their own pre-understanding regarding the subject, as parents, as registered nurses and as specialist nursing students. However, as the coding process was kept close to the text, verbatim and without any interpretation during the coding process (21), interpretations colored by researcher pre-understandings was avoided. Each interview was labelled with a number (Interview xx) and units of text and quotations were coded, close to text, with as many words as possible [21]. Every quote, text unit and code were labelled with the same number as the interview from which it originated. All the codes from the first authors were then compiled, compared, and sorted into groups with similar meaning. A digital whiteboard was used to move the codes around to get a clearer view on where they belonged. This was done by the first authors jointly. Every group was then given a headline that represented the content, and this is how the sub-categories were made. Example of this can be seen in Table 1. After doing that the generic categories were created by grouping the sub-categories. The quotes from the interviews were used to support the analysis of the text and the quotes that are presented in the results are labelled with each interviewee’s number. The first authors contributed equally during analysis. 12 subcategories and four generic categories were developed through the analysis, all emerging into one main category which are illustrated in Table 2.

**Table 1:** Analysis

Citations in the text	Codes	Sub-category	Generic category
” I felt angry” [9] “I guess I got pretty angry” [2] “I went home and cried” [6]	Anger Anger Sadness	Strong emotions and reactions	A hard time coping

## Results

The result of this study showed how talking to parents about their child’s weight is “An Emotionally Loaded Subject” as the parents reacted with strong emotions when receiving the information about their child being overweight. It was hard for them to accept that it was true, and they questioned the measuring tools as well as whether the extra weight was unhealthy or not. They were afraid that the conversation might hurt the children and feared that the children would be bullied. They also wanted to protect the children from the norms in society and how the CHC-nurse approached the subject was significant for the outcome of the conversation.

**Table 2:** Results

Sub-categories	Generic categories	Main category
Strong emotions and reactions	A hard time coping	An emotionally loaded subject
Doubting the assessment		
Questioning whether the weight is unhealthy		
Child participation in the conversation	A desire to protect the children	
Societal Norms		
Fear of the effects of exposure to overweight stigmatization		
To judge oneself	Changes of Parental Perspective	
To feel judged by others		
To be responsible		
The Encounter	Trust - a mediating factor	
The trust in the CHC-nurse		
The support		

### A hard time coping

Parents reacted strongly to the initial shock. They were inclined to doubt whether the assessment of the child was correct and questioned the health risks.

#### Strong emotions and reactions

The information about their children being overweight came as a shock for the parents, particularly those who had previously struggled to achieve the desired weight according to the growth chart. Those, whose children had always been on the heavier side, felt surprised when it was suddenly presented as a problem. The conversation awoke many feelings in the parents, and they described it as difficult to hear and tough to accept. They felt angry, irritated, or defensive and one dismissed the information completely. They also expressed feelings of sadness and one said that she was so sad that she went home and cried afterwards. The sensitivity of the subject was highlighted and when parents were overweight themselves, they expressed that their own experiences made it extra sensitive and difficult to hear.

#### Doubting the assessment

The parents did not agree with the fact that their children were being overweight and explained that it was difficult to take the situation seriously, and the health care system’s perspective on overweight was described as obsolete and outdated. They described that there is too much faith in the BMI-scales and the growth charts, which they felt did not take aspects such as muscle mass into account, and therefore had too much impact in the assessment of the child.

#### Questioning whether the weight is unhealthy

The question whether the extra weight was fat, or muscles occurred frequently, and since the overweight was not an obstacle in the children’s lives, some questioned whether the overweight was actually a health concern.

*He weighs a lot*,* yes*,* but is it that unhealthy in his case? Does he have that*,* like*,* unhealthy overweight that leads to*,* like*,* diseases? (Parent 6).*

The parents dismissed or questioned the information regarding the overweight since their child, to them, had no visible overweight, and alternative words were used to describe their children’s weight status, such as big, heavy, chubby, rounded, and solid. They explained that children grow unevenly, and they consider it normal for a child to first gain weight and then grow taller. The parents also expressed that their children looked like their relatives, and that it is not surprising that they have this kind of physique. They questioned whether their lifestyle habits actually have had any impact on their children’s weight situation since they, in their opinion, already had healthy habits. They also doubted that changing their habits could make any difference, due to the genetic nonadjustable composition. They also believed that the weight would resolve by itself in time.

### A desire to protect the children

The main concern of the parents was to protect their child from any potentially negative consequences, such as a damaged self-image, self-stigmatization and bullying in the future.

#### Child participation in the conversation

There was a fear among the parents that the children participating in a conversation about being overweight would affect the children negatively and cause eating disorders. The parents highlighted that they are aware that being overweight is unhealthy and they understood why the CHC-nurse raised the issue. However, the fear of eating disorders and that the conversation would make the child connect food and weight in a negative way was described as outweighing the risks of being overweight.

.*I think that being overweight is absolutely not good*,* it’s harmful*,* but anorexia actually kills… (Parent 9)*.

The parents worried that the children’s participation in the conversation might hurt them or make them lose their self-esteem and change the way they look upon themselves and their abilities. They feared that their children would develop unhealthy body images, start thinking about their body size or start identifying as overweight.

*I don’t want my little girl to start thinking about whether her body is too fat or not… (Parent 9)*.

The parents explained that children understand from an early age that being overweight is not considered a good thing, and they did not want their children to be present when addressing this subject. They expressed a theoretical possibility of a child participating in the conversation, if it was done under the right conditions, like a lifestyle conversation at the child’s level of understanding, but they still believed that their children were too young to be a part of these conversations and did not appreciate that they were.

.*what made me most angry was the fact that she brought it up in front of my daughter*,* I mean she’s 3 years old […] children understand more than you think… (Parent 2)*.

#### Societal norms

The parents explained that today’s cultural norms promote being as thin and fit as possible and children are affected by this both directly and indirectly. Because of that they desired to not expose their children to this and to attempt to counteract this by different means. They either avoided talking in terms such as tall, short, fat, and thin, or used the words but without attaching any value to them. It was also described that they tried to attribute positive values ​​to words like “fat” so their children would not associate it with something negative. If they talked about their bodies with their children at all they tried to focus on what the body can do and what it is good at instead of how it looks. The parents did not wish to talk about certain food as something bad or something that should be avoided and explained that if they discuss food with their children, it is about which food is good for you and will make you strong and healthy. As far as possible they use value-neutral words regarding food and exercise, and they avoided talking about weight and food together, and they did not appreciate it when these subjects came up during the conversation at the CHC.

.*” If you eat too much of this you will get fat”*,* and I was like*,* that isn’t something that a 3–5-year-old needs to hear about*,* like*,* even if it is true… (Parent 4)*.

#### Fear of the effects of exposure to overweight stigmatization

The parents were also concerned about the future and how others would treat their children because of them being overweight. They feared that their child would be left out or feel that they did not belong or that the child would suffer from mental illness and poor self-esteem. The parents also feared that being overweight would lead to the child being bullied and those who were overweight in their own childhood felt particularly afraid about being bullied and explained that the treatment you receive varies greatly depending on your body shape.

### Changes of parental perspective

The parents experienced self-doubt and a lack of confidence, not only did they judge themselves but also felt judged by others.

#### To judge oneself

Having this conversation regarding their children being overweight made the parents question their parenting skills and they expressed feelings of guilt and shame. They were afraid to have done something wrong or to have made the wrong decisions or harmed their child. The parents explained that they always try to do what is best for their children and they thought they did it right, and now it felt like a failure when that was not enough.

One parent shared that she struggled with being overweight herself, and always had as a goal that her children should not get there, and now when they were, it felt like a failure. Even though she knows it was not an actual failure, the sensitivity in the subject made it feel more personal than it was.

#### To feel judged by others

The parents expressed that the conversation made them feel judged and criticized. One of them explained that every health check-up the child goes to feels like an assessment of you as a parent, and if something is not perfect it feels like criticism against their parenthood.

*…I think that the instinctive feeling is*,* or was for me at least*,* that I did not do a good job*,* and she judged me for that. (Parent 9)*

The parents not only expressed feelings of being judged by the healthcare system, but by society itself and there was a fear that other people would question how they took care of their children and what habits they had.

.*it feels like people think you give them candy every day*,* and just eat unhealthy food and never exercise (Parent 1)*.

It was also expressed that it is easy to have opinions about what other parents do, but when you find yourself in the same situation, it is not as simple as it seems.

#### To be responsible

The parents felt divided since they were responsible for their children’s habits and therefore causing the problem and were now responsible to put it right. It was perceived as against the parental instinct to limit the child’s food intake, and it felt difficult to justify this to the children. It was described as difficult when they could not give their children more food when they asked for it, especially since they knew of others who had trouble getting their kids to eat at all. For those who were separated from the other parent, it felt difficult to respond to questions about the child’s food and eating habits since they could not speak for the other parent. They wished the other parent should have been contacted as well, even those cohabitating, since it was described as hard to pass on information and advice, especially if the parents had different opinions on what is right for the child.

### Trust-a mediating factor

The parents’ relationship with their CHC-nurse and how she treated them during the encounter affected their acceptance and how well supported they felt in the situation.

#### The Encounter

The parents highlighted the importance of respectful healthcare encounters and to establish a good relationship with their CHC-nurse. Those who felt they were treated nicely during the conversation found it easier to handle the situation while parents who had a bad experience wished for more support. The CHC-nurse was described as harsh and plump while telling them about the child being overweight and they felt like she did not take the sensitivity of the situation into account. They perceived the CHC-nurse as too straightforward and insensitive which made them feel as if they were caught off guard. They wished for more support and encouragement, and they would have liked to hear some calming and reassuring words rather than how bad it was. However, those who did receive reassurance such as ‘*it will probably even out’* said that even though it was comforting to hear, it felt like the CHC-nurse mitigated the problem or was afraid of stepping on someone’s toes. The parents also expressed that the CHC-nurse seemed uncomfortable and had difficulties raising the subject, which made the conversation feel awkward.

A neutral and matter-of-fact approach was appreciated by the parents, and it was preferred when the nurse was calm and educational. It was described as positive for those who felt like the CHC-nurse showed understanding for the sensitivity of the subject and when the CHC-nurse seemed responsive to the parents it was easier for them to receive the message. Sensitivity and humility were requested by the parents, while lack of empathy was described as a problem in the situation and better treatment was wished for.

#### The trust in the CHC-nurse

Whether the parents trusted the CHC-nurse or not was described as significant for the outcome of the conversation. It was described as hard to have the conversation with a CHC-nurse they did not trust. It was difficult to trust a CHC-nurse in this conversation when there was a previous history of being forgotten or double booked, and the trust was also affected by how child friendly the room was. When there were no toys to play with and the child did not like to be there, neither did the parents. One parent described that she felt unwanted during the entire appointment as the CHC-nurse sighed heavily in relation to every moment of the check-up and did not seem to want them there at all.

The situation was also affected by the advice they got. Non-evidence-based advice affected their faith in the CHC-nurse in a negative way, and one parent found it questionable when they got advice to make more unlikable food to try to get their child to eat less. The parents also felt like the CHC-nurses jumped to conclusions regarding their lifestyle habits and assumed that they did something wrong. They said that the CHC-nurse showed no interest in what kind of lifestyle habits they had, and instead instantly pointed out what to do differently, which felt extra unwarranted for those who never met the CHC-nurse before.

Those who never met the CHC-nurse before had a hard time trusting her at all, and to have this particular conversation with a CHC-nurse they did not know was not appreciated.

It was the first time she met us, and sure, she does not know at all… then maybe you should not just assume that people eat junk food and sit still… (Parent 9).

Those who trusted their nurse from the start described that the faith they already had in the nurse affected the situation positively, and even though it was a delicate situation it was not perceived as a negative experience. One parent explained that they have had the same CHC-nurse since the child was born which had created a trusting relationship between them, and if someone else had given the information, it would not have been received in the same way.

.*she [the CHC-nurse] really understands the problem in a different way[…] it feels like*,* the doctors who come at these check-ups who just go in and point[…] it doesn’t get personal then*,* it’s like this “okay you’re talking about a chart*,* but I’m talking about my child”*,* it’s a bit different. (Parent 8)*

#### The support

What support the parents were offered from the CHC-nurse varied greatly, some received dietary advice, follow-ups and lots of information regarding being overweight while others recived none at all. It was perceived in different ways since some did not want any information at all while others wished for more. It was also described as difficult to receive advice on what measures to take while still being in shock. Some did not want either advice or follow-ups, although many were offered extra weight checks or a referral to an obesity unit, which they had different opinions on. Some went there with their children while others felt it was an overreaction from the CHC-nurse.

.*that we would get a follow-up*,* because it could also be something medically wrong. Like this*,* yes*,* but it’s also pulling the trigger all at once… (Parent 2)*.

## Discussion

The results of this study indicated that talking about their child being overweight was a sensitive topic for the parents and they reacted with strong emotions, which confirms previous research [11]. In accordance with other studies [12, 15] the parents struggled with acceptance, which could be explained by the fact that they did not consider their children being overweight and had their own ideas regarding how children grow. Genetics was believed to be a main cause for being overweight, which is consistent with previous research [15–17], and there were questions whether it was possible to overcome genetic factors. Even though it is known that some children gain weight more easily, according to a large study [22], the primary factors regarding treatment of being overweight were environmental, social and behavioral, rather than genetic. The parents blamed themselves, felt guilty and questioned themselves, and some felt like they had harmed their children, which was similar to other studies [12, 18]. It was difficult for the parents to understand how it turned out this way for their children and they felt there was nothing they could have done to prevent it. This differs from another study where fathers to a greater extent could see their own participation in how it came to this and what could have contributed to their children being overweight [23]. In light of this, CHC-nurses might benefit from having the genetic predisposition in mind, as well to have an individual approach and be careful not to blame the parents but help them make adjustment that suits the family.

The parents wished to protect their children from the consequences a conversation about them being overweight might lead to. They did not want the children to be present in the conversation at all, which agrees with another study [11]. They feared it might negatively affect the children’s self-esteem, cause unhealthy thoughts regarding food and weight or cause eating disorders. They wished to use neutral terms while speaking about weight and food and they would rather not speak about it at all while their child was present, and they did not like it when the CHC-nurse did. This could be understood by the *body positive movement* [24], where previous research shows that a body positive approach might have a good influence on children’s self-image and their view on food and weight [25]. Even though a body-positive approach might benefit the children’s self-image, in this case, it conflicts with the medical perspective where being overweight is, or might be, a health risk if not prevented [2].

The parents felt accused, questioned, and judged in the conversation regarding their child being overweight, and perceived it as criticism of their parenthood. They also felt guilty and judged themselves. This might be a result of the prevailing overweight stigma, where parents to children who are overweight are often exposed to associated stigmatization which is surrounded by presumptions regarding how they take care of their children [18]. This shows the importance of the CHC-nurse having the sensitivity of the subject in mind and to be caring, respectful and responsive when raising the subject to avoid stigmatization.

The parents questioned the validation of the measuring tools since the child being overweight was not visible, and whether extra weight was unhealthy or not. This can be considered an obstacle regarding the work preventing children being overweight and more generally obesity in society, in line with a previous study [17]. These findings suggest that there seem to be a perception among parents that when talking about excess weight in childhood it is about how the children look, rather than the health risks associated. There might be a gap of knowledge, where CHC-nurses would gain by educating parents about the measuring tools and how children are expected to grow, preferably before a weight issue occurs. It might also be beneficial for both CHC-nurses and parents if the CHC-nurses, during conversations regarding being overweight in childhood and the associated risks clarify that the conversation has nothing to do with how the children look and the risks remains whether the overweight is visible or not. CHC-nurses might also benefit from using neutral terms regarding food and exercise to decrease the risk of the children’s self-esteem being affected [25].

The parents felt like the CHC-nurse seemed uncomfortable talking about the subject which agrees with previous research [10]. They said it felt like she raised the subject without full concern for the sensitivity of the parent or the child, which could be understood by Sjunnestrand et al. [9] where CHC-nurses felt like they were obliged to raise the subject whether parents seem receptive or not. If some CHC-nurses feel uncomfortable, it might be one reason why parents in this present study had a negative experience. It is possible that raising the subject without responsiveness, and jumping to conclusions regarding their lifestyle habits, might create a wall between the CHC-nurse and the parents [26], which make the parents less likely to be motivated to make any changes. This highlights the importance of CHC-nurses building a caring relationship with the parents. If the relationship is poor and the CHC-nurse is perceived as uncaring [26] the parents might not be receptive to the information regardless of what the CHC-nurse does, therefore the whole purpose of guarding the child’s overall health through growth monitoring in line with the nurses’ work description [6] will have failed. This might lead to absence of changes in the family’s lifestyle habits.

This result also indicates that a pre-existing caring relationship is not necessarily affected negatively by talking about being overweight in childhood, which was something CHC-nurses feared in previous research [9]. The fact that the conversation was not perceived as negative when there was a caring relationship and the parents trusted their nurse, has also been previously shown [8]. It so appears that having a caring relationship might be a prerequisite when it comes to talking about being overweight in childhood. This could be understood by what Halldórsdóttir [26] explains as the bridge. When a bridge is built between the CHC-nurse and the parents, and the CHC-nurse is perceived as caring, the conversation might feel empowering [26] and the parents might get motivated to make changes. If that is the case, the CHC-nurse does not need to be afraid to bring up the subject as the trust between them will help to overcome any challenges that might arise during the conversation about the child’s weight. At the same time the CHC-nurse might benefit from showing understanding and sensitivity towards the parents’ concerns and be careful regarding use of terminology and when and how to talk about children’s weight, and to ask open questions regarding lifestyle habits rather than just assume they do something wrong. By doing so she might be perceived as careing and it is possible that the conversation might empower the parents [26] rather than offend them.

### Limitations and strengths

This study has some limitations, first the parents were interviewed after being sought via social media. Since all participants volunteered to participate there could be a risk of sampling bias [20] which might decrease the transferability since parents might have chosen to participate because they had strong feelings they wanted to express regarding the subject. However, since similar studies [11, 12] where participants were recruited in different ways show similar results which strengthen the trustworthiness of the result.

Second, nine parents were interviewed, and even though a greater number might have enriched the result further and increased the credibility and transferability [20], more interviews would have contributed to more data than what was manageable considering the time limit when performing the study. During the last couple of interviews, the same kind of experiences were described and nothing new came up, hence, saturation was achieved, which is considered a strength [20].

There are some strengths in the study. Firstly, since all the coding was done separately by the two first authors, and turned out to be similar, it decreased the risk of preconception bias and contributed to the trustworthiness and credibility in the result. Second, the person participating in the pilot interview read the result and expressed recognition, even though her interview was not included, this further strengthened the credibility of the study [20]. Regarding transferability, internet is a broad platform with a variety in clientele when it comes to recruiting participants [20] and therefore it is possible that parents recruited in other contexts might have shared similar experiences since. Another study where parents were recruited through the CHC show a similar result [11], which might strengthen the credibility in this study, and the findings to be transferable, at least in a Swedish context. Furthermore, a study from Australia got a similar result [12] which suggests that it might be transferable to other western communities as well.

The main issue regarding trustworthiness within qualitative research is according to Elo et al. [27] within the preparation phase. To increase the trustworthiness in this present study their checklist for improving trustworthiness has been checked.

Using quotes in the result contributed to enrich and also authenticate the results since the reader can make their own judgment of whether or not the quotes are correctly assessed, also promoting confirmability [20].

### Clinical implications

This study increases the understanding of the parental perspective on children being overweight, the knowledge can be used to facilitate the CHC-nurses’ conversations about the child’s weight. This may increase compliance to given advice on lifestyle changes, to promote future health for the child.

Since the parents seem to lack knowledge regarding how children are supposed to grow and how the measuring tools work, it might be beneficial for CHC-nurses to introduce the growth charts as a routine from the beginning when the child is born, so the parents understand what it is for and how it is used. By doing that in every appointment, the parents get used to the growth chart and might not react so strongly when the CHC-nurse shows it when something deviates. This might faciliate the following conversation. If parents understand why they have to take measures regarding their children’s increased weight it might improve the compliance to the given advice.

The subject seems to be sensitive to the parents, hence the CHC-nurses might benefit from talking in neutral terms and not mention anything about the child being overweight while the child is present, and just talk in general about healthy lifestyle choices on the child’s level and then contact the parent again afterwards to have this discussion on a separate appointment or by phone.

## Conclusion

Based on the result of this study it seems like parents take the conversation about childhood overweight personally, and it could be perceived as being critical of their parental skills. In their perspective, the conversation was about how their children looked rather on their health, and since they did not consider their children being overweight, the conversation as well as the following measures seemed unnecessary. When CHC-nurses raised the subject, the parents felt judged, and they feared their children might get hurt in the process. This highlights the importance for CHC-nurses to be responsive towards when and especially *how* to talk about the subject, without blaming the parents or risk hurting the children’s self-image or self-esteem. Parents need time to process the situation before receiving advice and it might be beneficial not to talk about weight when the child is present, and focus should be on healthy lifestyle choices rather than weight.

## Data Availability

The author confirms that all data generated or analysed during this study are included in this published article.Consent to publish: Not applicable.

## Abbreviations

BMI: Body mass index
CHC: Child health care
GDPR: General Data Protection Regulation
WHO: World health Organization
